# Age Specific Models to Capture the Change in Risk Factor Contribution by Age to Short Term Primary Ischemic Stroke Risk

**DOI:** 10.3389/fneur.2022.803749

**Published:** 2022-02-17

**Authors:** Elizabeth Hunter, John D. Kelleher

**Affiliations:** ^1^PRECISE4Q Predictive Modelling in Stroke, Technological University Dublin, Dublin, Ireland; ^2^ADAPT Research Centre, Technological University Dublin, Dublin, Ireland

**Keywords:** stroke, predictive modeling, machine learning, epidemiology, risk

## Abstract

Age is one of the most important risk factors when it comes to stroke risk prediction. However, including age as a risk factor in a stroke prediction model can give rise to a number of difficulties. Age often dominates the risk score, and also not all risk factors contribute proportionally to stroke risk by age. In this study we investigate a number of common stroke risk factors, using Framingham heart study data from the NHLBI Biologic Specimen and Data Repository Information Coordinating Center to determine if they appear to contribute proportionally by age to a stroke risk score. As we find evidence that there is some non-proportionality by age, we then create a set of logistic regression risk models that each predict the 5 year stroke risk for a different age group. The age group models are shown to be better calibrated when compared to a model for all ages that includes age as a risk factor. This suggests that to get better predictions for stroke risk it may be necessary to consider alternative methods for including age in stroke risk prediction models that account for the non-proportionality of the other risk factors as age changes.

## 1. Introduction

Globally stroke is one of the leading causes of mortality. In the US the annual direct medical costs for stroke in 2012 was 71.55 billion US dollars (1). This cost, however, only takes into account the direct costs and does not consider years lost to work or other societal burdens that are caused by stroke, so the actual cost is much higher. One way to reduce the overall burden of stroke on society is to find better ways to prevent stroke. Currently, one of the methods used for stroke prevention is to identify patients with a high risk of having a stroke based off of certain risk factors. This identification is often done through risk prediction models, such as SCORE (2) and the Framingham heart study model (3, 4). Once identified a patient can be advised to adjust their behaviors or be treated with appropriate medications to lower their risk of having a stroke.

The risk factors used to predict stroke can either be modifiable or non-modifiable (5). One of the most important non-modifiable risk factors is age, the older a person is the more likely they are to have a stroke (6). Although its important to take age into consideration when predicting stroke risk, it can also, lead to a number of complications. As age is non-modifiable, if an individuals stroke risk is high solely because of their age, it can lead to unnecessary stress as they cannot change their age. Conversely, having a low risk score due to age might make an individual complacent despite having other risk factors for stroke that will lead to high risk as they age. Approximately two thirds of US adults who have low short term stroke risk have been shown to have a high lifetime risk (7). Additionally, age is such a strong predictor of stroke, if it is included as a risk factor in a model it can sometimes dominate the risk score and thereby can lead to under prediction in the younger age groups and over-prediction in the older age groups (8).

When age is considered as a risk factor, this assumes that the contribution of the other risk factors in the model are proportional by age, in other words that their contribution to risk does not change with age. For example, under the assumption of proportionality by age, having diabetes as a 45 year old produces the same level of stroke risk as having diabetes at 80. This assumption is particularly important when looking at short term risk where individuals are likely to stay in an age group for the whole prediction period. This may be a problem as it is likely that risk factors differ between the young and old (5). Another example, of why the assumption of proportionality by age does not hold is the impact of age on sex as a risk factor. In the study by (9), they showed that younger women have lower short term risk of a stroke than men but as they age, this switches and women have higher short term risk than men. Thus, the contribution of sex as a risk factor should be different based on an individual's age.

In this paper we aim to answer two questions: (1) is there evidence that the contribution of stroke risk factors to an individual's 5 year stroke risk are non-proportional by age, and (2) if there is evidence that risk factors are non-proportional by age, how can we create a risk model to better capture this non-proportionality when predicting an individual's risk of stroke. To answer these questions we investigate if there is a difference in risk factor contribution to stroke risk by age to short term stroke risk, and to account for any difference we propose creating a stroke risk prediction model that is made up of four separate models, each covering a different age group. We first discuss the data used, then we present a statistical analysis of the data looking specifically at risk factors by age group. Finally we create multi-variable logistic regression stroke risk prediction models, comparing a model created using the full age range with a set of age specific models created by breaking the data set into different age groups.

## 2. Materials and Methods

In the following sections, we discuss the data set used for our analysis, the modeling data sets we selected from the full data set and finally our methods for analyzing the data and modeling stroke risk.

### 2.1. Data

We use data from the Framingham Heart Study combining the Framingham-Cohort, Offspring, Third Generation, OMNI 2, and New Offspring cohorts from the NHLBI. The Framingham Heart Study is a longitudinal study that began in 1948 that sampled both men and women in Framingham, Massachusetts with the goal of studying the incidence and prevalence of cardiovascular disease and its risk factors over time. Participants recruited into the Framingham Heart Study undergo clinical examinations and complete lifestyle and medical history questionnaires at regular intervals. The Framingham-Cohort data set recruited 5,209 men and women, aged 28–62, between 1948 and 1952, and has data for 32 clinical exams per subject through 2018 (10). The Framingham Offspring data set is made up of 5,124 men and women, between the ages of 5 and 70, who were recruited as offspring of the original Framingham cohort or spouses of offspring, and has data from 9 clinical exams per subject through 2018 (11). The Omni 1 cohort is made up of 507 men and women of African-American, Hispanic, Asian, Indian, Pacific Islander, and Native American origins, who were recruited in 1994 and were residents of Framingham or the surrounding towns. There is data from the first four clinical exams per subject from the Omni cohort (11). The third generation cohort is made up of 4,095 men and women, ages 19 plus with at least one parent in the Framingham Offspring cohort, and has data from the first 2 clinical exams per subject through 2018 (12). The New Offspring Cohort enrolled spouses of Offspring participants who were not already enrolled and had at least two biological children participating in the Generation three cohort. The new offspring cohort recruited 103 men and women (12). The Omni 2 cohort enrolled 410 participants who were ethnically diverse compared to the other cohorts which are predominately white. Some of the Omni 2 cohort are related to those who participated in the Omni 1 cohort while others are unrelated (12).^1^

As we are looking at short term risk, we do not look longitudinally at an individual but instead break down the data set into separate records for each clinical exam. This means that the same person might appear in our data set more than once. Across the cohorts there are 113,714 clinical exam records. To look at short term ischemic stroke risk we created a flag for clinical exams of individuals who suffered an ischemic stroke within 5 years of that clinical exam. For the purpose of our study we are only looking at the risk of ischemic stroke so we do not consider anyone who has a hemorrhagic or unknown type of stroke.

We remove any clinical exams from the data set where there is missing data for any of the continuous risk factors we are considering: systolic blood pressure, diastolic blood pressure and total cholesterol. For BMI, if there is no value for BMI in a given data collection year but a weight for that year we use the height collected in the first data collection phase and calculate the individual's BMI with their height and current weight. Where there is missing data for our categorical variables as most categorical variables are to identify a condition or treatment we consider a missing variable equivalent to not having the condition or not smoking.

#### 2.1.1. Modeling Data Set: Age Groups

In order to assess the non-proportionality of risk factors by age, we separate the data set into four data sets based on age: less than 50, 50–59, 60–69, and 70 plus. As the data is greatly unbalanced we select two controls for each stroke. The controls are matched on age and cardiovascular disease status and are selected from the set of clinical exams where the individual examined did not suffer a stroke within 5 years of the exam. We do not control for those who have had a stroke outside of our time window, thus a clinical exam in the controls might be for an individual who suffered a stroke 6 years after the clinical exam or 20 years after the examination, meaning the individual's overall stroke risk might still be higher than the control clinical exams for individuals who have not had a stroke during their lifetime. This down sampling of non-strokes resulted in a data set of 113,714 clinical exams, one third of which have had a stroke within 5 years. Note that, the 1:2 (stroke:non-stroke) ratio is maintained across the four age groups. When modeling on all age groups with age as a risk factor we combine the four age groups data sets to create a single data set that contains all ages.

### 2.2. Statistical Analyses and Modeling

The first part of our study is an analysis that assesses whether there is a statistically significant difference in the data for risk factors by age group. This analysis is done to determine if risk factors are non-proportional by age. This statistical analysis is itself composed of three parts. First, we present descriptive statistics of the full data set and the modeling data sets. We then examine variation in each risk factor across age groups where for each age group cohort we only consider those people who had a stroke, and finally we examine risk factors variation within each age-group between those who have had a stroke and those who have not.

Looking at risk factors across age groups we aim to determine if we see a difference in the distributions of a given risk score across the age groups. Different distributions would suggest that a risk score might contribute non-proportionally to an individual's risk score by age. There are two data type for the possible risk factors in the model: continuous and categorical. As such there are different statistical tests that need to be considered for each type of data. For each of the continuous risk factors (systolic blood pressure, diastolic blood pressure, total cholesterol, BMI, and cigarettes per day) we use a Kruskal-Wallis test to assess the variation of the risk factor across age groups. The Kruskal-Wallis test is a non-parametric test that determines if the medians from more than two independent groups are the same (13). If the test is significant (*p* < 0.05), than the distribution of the risk factor is not the same for all age groups. Note that for the cigarettes per day risk factor we consider two encodings of the variable: one for all individuals and one for only smokers. We do this because when all individuals are included in the statistical tests for the average number of cigarettes smoked per day, all of those individuals who do not smoke will have a value of 0. This could bias the results, thus we also look at the distribution of cigarettes per day in only those who smoke to determine if there is a difference in the number of cigarettes smoked for those who do smoke and have had a stroke vs. those who do smoke and have not had a stroke. For the categorical variables (sex, smoking, atrial fibrillation, diabetes, high blood pressure treatment) we use Pearson's Chi-squared test, which is a non-parametric test that determines if there is a difference in the occurrence of a dependent variable based on an independent variable (14). As noted above, regardless of the statistical test run (Kruskal-Wallis for continuous or Pearson's Chi-squared for categorical factors), if the *p*-value for a test is significant than there is a statistical difference between the distribution of the risk factor across the age groups for which the test was run.

For the analysis examining risk factors across age groups we restrict the population of each age group to only those who are identified as suffering a stroke within 5 years of a clinical examination that was carried out when they were in that age group. This is done for two reasons. The first is that it will identify only the risk factors whose distributions differ across age groups for the population we are looking to predict, those who have had a stroke. The second reason is to meet statistical assumptions. One of the assumptions of the Pearson's Chi-squared test is that a single individual can only contribute data once to the data being analyzed (14). In our sampling method, we allow for the same individual to be included multiple times, once for each clinical exam. However, as only the first stroke is recorded in the data set an individual can only appear once in the stroke population of our data.

Once we defined a data set for each age group composed of those who suffered a stroke within 5 years of a clinical examination within the age group, we then take each risk factor in turn and run a statistical test on this data set to assess whether the variation of that risk factor across the age groups was likely caused by random variation or represents a statistically significant variation. If the *p*-value returned by the statistical test for a given risk factor is less that 0.05 we will take this to signify that there is a statistically significant variation in that risk factor across age groups among those who have had a stroke. Such a finding would provide evidence that the contribution of that risk factor to stroke risk is non-proportional by age and this would justify investigating further the differences in risk factors by age and signal whether there is likely non-proportionality in risk factors by age.

After looking across age groups we then look within age groups comparing those who have had a stroke within 5 years of a clinical examination carried out when they were in that age group and those who did not have a stroke within 5 years of their clinical examination within the age group. In this analysis we assess: (i) which risk factors might make important contributions to the risk for an individual in a given age group, and (ii) if the risk factors that might be important vary across age groups. We carry out a separate statistical test for each risk factor in each age group to determine if the variation of a risk factor between those within the age group who have had a stroke and those within the age group who did not have a stroke is statistically significant. If there is a statistically significant difference in the distribution of a risk factor between those who have had a stroke and those who have not had a stroke then the risk factor may be important in calculating stroke risk for people within that age group. This in itself would be an important finding. Furthermore, with respect to our main research question (1), if we observe that for a given risk factor there is a statistically significant variation of its occurrence between the stroke and non-stroke population in some age groups but not in others this would provide further evidence that the contribution of factors to stroke risk is non-proportional by age. For the analysis of risk factors within age groups, again, we have two types of variables continuous and categorical. For the continuous variables we use a Wilcoxcon Rank Sum test which is used to test if two samples are likely derived from the same population (15). Similar to the across group analysis, for the analysis of the categorical variables within groups we use a Pearson's Chi-squared test. As before, irrespective of the type of test run, if the statistical test run for a given risk factor within an age group returns a significant *p*-value this would signify that there is a difference between the distribution of that risk factor for those who have had a stroke and those who have not within that age group.

After the statistical tests are completed, the second part of our study focuses on modeling and aims to answer the question how can we create a risk model that predicts an individual's stroke risk to better capture this non-proportionality in risk factors by age. In our risk analysis we use logistic regression models. While there are other types of models that are utilized for stroke risk prediction, for example survival analysis, we choose logistic regression as in our analysis we focus on whether a stroke has occurred within a time window (rather than when in that time window a stroke occurs). A logistic regression risk model takes one or more risk factors or features from an individual and predicts that individual's risk of an event occurring within a predefined time-window, in this case stroke within the next 5 years. We run a multi-variable logistic regression and compare a single multi-variable regression that includes age as a risk factor against a set of multi-variable models that do not explicitly consider age as a factor but where each model in the set is fit to a specific age cohort and taken together the set of age cohort specific models covers the age spectrum. For any given individual their stroke risk is predicted using the model trained on the age cohort that corresponds with the individual's age. The other risk factors we consider in the models are sex, systolic blood pressure, diastolic blood pressure, BMI, average cigarettes per day, atrial fibrillation, and diabetes. For risk factors that are continuous: age, BMI, and systolic and diastolic blood pressure we scale the variables by standardizing (subtracting the mean and dividing by the standard deviation). This does not change the predictions of the model but allows us to interpret the coefficients of all the variables on a similar scale.

In comparing the multi-variable models (the single model including age and the ensemble of age cohort specific models) we look at the changes in coefficients, the predictive ability of the models using discrimination measures and the calibration of the models. Each of our logistic regression models are designed and trained to predict a personalized stroke risk score for an individual based on their values for the risk factors the model considers (e.g., sex, smoking, systolic blood pressure, and so on). Although the models function by making predictions on a per individual basis, they are evaluated based on their performance across a group of individuals. In other words, model performance is reported using aggregate statistics calculated across the model's performance on a number of individual cases (e.g., the average model accuracy across a sample of individuals that were not included in the dataset the model was fitted to during training). While discrimination gives a measure of the predictive power of a model or how well the model is able to fit to the data, calibration is a measure of the difference between the estimated and the true risk and gives an idea of how the model will fit to a different data set (16). Looking at calibration, in addition to the more commonly used discrimination measures, is important as it can help to identify models that on the whole discriminate well but that do not predict well for certain groups, for example older or younger patients (16).

To calculate the discrimination and calibration measures we use k-fold cross validation with 10-folds to estimate average of each metric we describe below. We look at a number of different measures for discrimination, focusing on the F1 measure, the AUC (area under the receiver operating characteristic curve), and the accuracy of the model. The F1 measure is the harmonic mean of precision (how often a model makes a positive prediction that is true) and recall (the true positive rate). The AUC plots the true positive rate against the false positive rate as the prediction threshold of a model and ranges from 0 to 1. Accuracy or the classification accuracy is the portion of predictions that are correct (17). For calibration we use the Spiegelhalter's *p*-value and the Hosmer-Lemeshow test designed to predict goodness of fit. The Spiegelhalter's *Z*-test separates the calibration aspects out of the Brier Score^2^ (18). If the *p*-value for the test is significant this suggests that the model is not well-calibrated (16). The Hosmer-Lemeshow test measures calibration by looking at goodness of fit. The test divides the data into a set number of groups and the test statistics is calculated by comparing the number of events in each group with the expected number of events in each group determined using the predicted probabilities from the model. Similar to the Spiegelhalter's *p*-value, if the *p*-value for the Hosmer-Lemeshow test is significant the model is assumed to be not well-calibrated (18).

As a final analysis of the models we look at the ranking of importance of features in each multi-variable model (the single model including age and the ensemble of age cohort specific models) to determine if this changes across the different age models. This allows us to determine which risk factors are the most important in each model. A difference in the order of the importance of features across the different age models, will provide additional evidence that contribution of risk factors vary by age group and thus there might be non-proportionality of risk factors by age. To calculate feature importance we use the *varImp* function from the Caret package in R that calculates feature importance for a linear model, such as logistic regression, by comparing the absolute values of the t-statistic for each model parameter (19). The contribution of each feature to the logistic regression model is calculated using the absolute value of the t-statistic for each feature.

## 3. Results

### 3.1. Data Description

Before performing any statistical analyses we first look at descriptive statistics of the data set. The following sections present these descriptive statistics for the full data set used and the modeling data sets.

#### 3.1.1. Full Data Set

Table 1 gives the number of strokes that occur within 5 years of a clinical exam for the whole data set and by age groups. We also include the counts of those who had a stroke at any point in their lifetime and number of clinical exam records without a stroke in 5 years or in the patient's lifetime. From the table, we observe that there are more lifetime strokes than strokes within 5 years of a clinical exam. The difference between the value for lifetime strokes (14,983 in the whole data set) and strokes within 5 years (2,114 in the whole data set) are those strokes that occur outside of a 5 year window from a given clinical exam and these strokes are not used in our analysis.

**Table 1:** Ischemic strokes by age group.

	**Total**	**Less than 50**	**50–59**	**60–69**	**70 plus**
Stroke in 5 years	2,114	106	277	586	1,145
No stroke in 5 years	111,600	36,709	28,510	24,540	21,841
Lifetime stroke	14,983	3,821	4,066	3,929	3,167
No lifetime stroke	98,731	32,994	24,721	21,197	19,819

Although all do not end up in our final models, we consider the following 10 risk factors: Systolic Blood Pressure, Diastolic Blood Pressure, Total Cholesterol, BMI, Sex, Smoking, Average cigarettes per day, Atrial Fibrillation, Diabetes, High blood pressure treatment.

Table 2 shows the distributions of the continuous risk factors across the data set for the whole data set, those who have had a stroke in 5 years and those who have not had a stroke. In the table, we show the minimum, median, mean, and maximum values and the number of missing values for each risk factor. From the table, we can see that the distributions from those who have had a stroke in 5 years and those who have not had a stroke in 5 years appear to be different across the risk factors, with the distributions for those who have not had a stroke similar to the distribution for the whole data set. Most noticeably is the large difference between the median and mean systolic blood pressure in the stroke and no stroke groups.

**Table 2:** Distributions of continuous stroke risk factors.

	**Minimum**	**Median**	**Mean**	**Maximum**	**Missing**
**Systolic blood pressure**					
Total	52	130.0	134.1	390.0	1417
Stroke	79.0	150.0	155.1	390.0	70
No stroke	52.0	130.0	133.8	383.0	1347
**Diastolic blood pressure**					
Total	25.0	79.0	78.9	163.0	354
Stroke	38.0	80.0	80.7	148.0	14
No stroke	25.0	79.0	78.9	164.0	340
**BMI**					
Total	12.0	25.8	26.4	66.6	1437
Stroke	14.0	26.4	26.9	53.6	55
No stroke	12.0	25.8	26.4	66.6	1382
**Total cholesterol**					
Total	27.0	212.0	214.4	1124.0	27906
Stroke	49.0	174.0	215.1	608.0	879
No stroke	27.0	212.0	214.4	1124.0	27027
**Cigarettes per day (only smokers)**					
Total	0	20.0	19.4	100	13,019
Stroke	0	20.0	20.1	60	175
No stroke	0	20	19.4	100	12,844

Table 3 shows the distribution of the categorical variables across the data and for each variable level counts for those who have had a stroke in 5 years and those who have not had a stroke, the percent of those with the level of the variable that had a stroke and counts of missing variables. From the table, we can see that while strokes seem to be evenly distributed across males and females and those who smoke, the percentage of those with atrial fibrillation, diabetes or high blood pressure treatment that have had a stroke in 5 years is much higher than the percentage of those who do not have the risk factor and have a stroke. This suggests that these variables might be significant risk factors.

**Table 3:** Distributions of categorical stroke risk factors.

	**Stroke**	**No stroke**	**Stroke percent**
**Sex**			
Male	958	48,936	1.9
Female	1,156	62,664	1.8
Missing	0	0
**Smoking**			
Yes	470	27,880	1.7
No	1,470	70,913	2.0
Missing	174	12,807
**Atrial fibrillation**			
Yes	431	3,578	10.8
No	1,683	108,022	2.0
Missing	0	0
**Diabetes**			
Yes	245	4,261	5.4
No	1,856	107,023	1.5
Missing	13	316	
**High blood pressure treatment**			
Yes	901	21,459	4.0
No	1,041	74,027	1.4
Missing	172	16,114	

#### 3.1.2. Modeling Data Set

After creating the age group data sets for modeling we look at the distributions of variables across the different ages. Table 4 shows by age group the median value for each continuous risk factor for those who have had a stroke in 5 years and those who have not had a stroke in 5 years. The table shows that there are some variables whose medians do not seem to change across age groups such as BMI or cigarettes per day. However, there are some variables, systolic blood pressure, and total cholesterol where the medians appear to change both by age and between the stroke and non-stroke group.

**Table 4:** Median value of continuous variables by age group and stroke or non-stroke outcome.

	**Less than 50**	**50–59**	**60–69**	**70 plus**
**Systolic blood pressure**				
Total	127.0	137.0	141.0	148.0
Stroke	135.5	145.0	149.0	153.0
No stroke	124.0	133.0	138.0	145.0
**Diastolic blood pressure**				
Total	82.0	84.0	80.00	73
Stroke	84.0	90.0	84.00	75.00
No stroke	81.0	82.0	80.00	72.00
**BMI**				
Total	26.1	26.57	26.71	26.28
Stroke	27.2	27.57	27.06	26.43
No stroke	25.6	26.20	26.55	26.16
**Total cholesterol**				
Total	222.5	234.0	224.0	188.0
Stroke	247.0	238.0	227.0	191.0
No stroke	213.0	233.5	222.0	186.0
**Cigarettes per day (only smokers)**				
Total	20.0	20.0	20.00	15.00
Stroke	21.7	20.00	20.00	20.00
No stroke	23.7	20.00	20.00	12.00

Table 5 shows the percent of clinical exams where the individual had a stroke in 5 years in each of the categorical variable categories. From the table, it can be seen that the percentage of individuals who have had a stroke in 5 years and who smoke, have atrial fibrillation or diabetes changes with age group. This signifies that there might be a difference in risk factors by age group worth investigating.

**Table 5:** Percent of clinical exams with an ischemic stroke in 5 years for the categorical risk factors.

	**Less than 50**	**50–59**	**60–69**	**70 plus**
**Sex**				
Male	31.1	32.8	38.2	38.7
Female	36.0	34.0	36.3	35.3
**Smoking**				
Yes	31.0	27.8	44.6	41.0
No	35.4	37.6	34.5	36.8
**Atrial fibrillation**				
Yes	43.8	65.6	78.5	86.1
No	32.6	31.9	34.5	30.6
**Diabetes**				
Yes	22.2	50.6	58.7	39.9
No	33.8	31.3	34.3	37.0
**Blood pressure treatment**				
Yes	47.6	37.2	40.8	38.5
No	32.0	32.5	35.3	36.0

### 3.2. Statistical Analysis to Test Non-proportionality of Risk Factors

#### 3.2.1. Comparing Risk Factors Across Age Groups

We first investigate if the distribution of risk factors varies across age groups to determine if the risk factors that contribute to an individual's risk of stroke changes across age groups.

Table 6 shows the *p*-values for the Chi-Squared tests (categorical variables) and Kruskal-Wallis tests (continuous variables) looking at the differences in risk factors across age groups in individuals who have a stroke within 5 years of a clinical examination. For the categorical variables (sex, smoking, atrial fibrillation, diabetes, and high blood pressure treatment) all of the *p*-values are much less than 0.05 showing that there is likely a difference in the distribution of risk factors in those who have had a stroke across age groups.

**Table 6:** Pearson's Chi-squared *p*-value for categorical variables and Kruskal–Wallis *p*-value for continuous variables by age group.

	***P*-value**
Sex	1.936e-06
Smoking	<2.2e-16
Atrial fibrillation	<2.2e-16
Diabetes	1.536e-07
High blood pressure treatment	<2.2e-16
Systolic blood pressure	7.619e-09
Diastolic blood pressure	<2.2e-16
Total cholesterol	<2.2e-16
BMI	0.0005
Cigarettes per day (all individuals)	<2.2e-16
Cigarettes per day (only smokers)	0.1071

All variables except for cigarettes per day in the smokers have at least one age group with a distinct distribution of the risk factor in those who have had a stroke. For smokers who have had a stroke the *p*-value is 0.11 which although is not significant at the 10% level is still small and could be considered as potentially showing differences in risk factor distributions across age groups.

The analysis in this section provides evidence that for each risk factor we might see some differences in the contribution to risk of stroke by age.

#### 3.2.2. Comparing Risk Factors Within Age Groups

Our second statistical analysis of risk factors is carried out within each age group to identify risk factors that have different distributions between those who have had a stroke and those who have not had a stroke.

Table 7 shows the *p*-values for the Chi-Squared tests on the categorical variables (sex, smoking, atrial fibrillation, and diabetes) within each age group. From the *p*-values we observe that we do get some difference in significance across the groups. Most notably we see no *p*-values that would be considered significant in the categorical variables for the less than 50 age group. This is likely due to small sample sizes.

**Table 7:** Pearson's Chi-squared *p*-value for categorical variables and Wilcoxon rank sum *p*-value for continuous variables by age group (*indicates simulated *p*-value due to small sample sizes).

	**Less than 50**	**50–59**	**60–69**	**70 plus**
Sex	0.497	0.777	0.441	0.058
Smoking	0.557	0.003	0.0002	0.136
Atrial fibrillation	0.522	0.0002	<2.2e-16	<2.2e-16
Diabetes	0.717*	0.0008	1.75e-11	0.345
High blood pressure treatment	0.226	0.356	0.03	0.163
Systolic blood pressure	1.19e-05	4.407e-11	<2.2e-16	9.492e-10
Diastolic blood pressure	0.003	7.041e-11	5.162e-13	1.534e-09
Total cholesterol	0.003	0.098	0.016	0.118
BMI	0.025	2.934e-05	0.150	0.058
Cigarettes per day (all individuals)	0.317	0.003	1.61e-05	0.067
Cigarettes per day (only smokers)	0.340	0.874	0.004	0.0001

In the other three age groups we see that there is a significant difference in the distributions of those who have atrial fibrillation and a stroke in 5 years vs. those who have not had a stroke across the three groups. Diabetes appears to only have a significant difference in the distribution between those who have had a stroke and those who have not had a stroke in the 50–59 age group and the 60–69 age group. Similarly, the difference in smoking between those who have and had not had a stroke is only significant at the 5% level in the two middle age groups. However, the *p*-value in the 70 plus group for smoking is 0.14 which although not significant at the 10% level is small enough that it should be considered as a factor that might impact the risk of stroke. The only age group where the difference in the distribution of sex between those who have and have not had a stroke is significant is in the 70 plus age group and the difference in the distribution for high blood pressure treatment is only significant in the 60–69 age group.

In Table 7, we also present the results of our statistical analysis of the differences in the distributions of the continuous risk factors for those who have had a stroke within 5 years of a clinical exam and those who have not had a stroke by each age group. From the table, we can see that for most of the continuous variables we see differences in the distributions between those who have had a stroke and those who have not had a stroke. For systolic blood pressure and diastolic blood pressure all four age groups have significant differences. Looking at total cholesterol, the three youngest age groups have *p*-values that are significant at least a 10% level and the 70 plus age group has a *p*-value that is 0.12 which is close to being significant at the 10% level and thus could be considered to have an impact on the risk of stroke. Similarly, for BMI the only age group that does not have a *p*-value significant at the 5 or 10% level is 60–69 and this group has a *p*-value of 0.15 which could still be considered small enough to investigate the difference in BMI between the groups.

For the cigarettes per day variable, it appears that in the 50–59 age group whether someone smokes might have an impact on their risk of stroke but the number of cigarettes that the individual smokes might not be as important. In both the 60–69 age group and the 70 plus age group, the differences in the distributions of cigarettes smoked per day are significant both for only smokers and when smokers and non-smokers are considered. This might mean that not only does smoking have an impact on an individual's stroke risk but that the number of cigarettes an individual in these age groups smokes might also impact an individual's stroke risk.

### 3.3. Multi-Variable Logistic Regression

In the previous section, we found through statistical analysis that there is evidence that the contribution of risk factors to an individual's stroke risk might be non-proportional by age. In this section we use multi-variable logistic regression models to develop a method to predict stroke risk for different age groups taking the non-proportionality into account.

#### 3.3.1. Model With Age as a Factor

Table 8 presents the coefficient for the multi-variable logistic regression model that includes age as a risk factor.

**Table 8:** Coefficients for the multi-variable logistic regression model including age as a risk factor (*indicates a scaled variable).

	**Coefficient**	***P*-value**
Intercept	−0.989	<2e-16
Sex	0.07	0.29
Systolic blood pressure*	0.16	3.8e-5
Diastolic blood pressure*	0.35	<2e-16
BMI*	0.08	0.016
Cigarettes smoked per day	0.02	3.5e-8
Atrial fibrillation	2.47	<2e-16
Diabetes	0.59	1.6e-8
Age*	0.18	2.8e-5

##### 3.3.1.1. Discrimination

To evaluate the model we look at both discrimination measures and calibration measures. Table 9 shows the discrimination measures when tested on all age groups together and on individual age groups. As the all ages measures are calculated using a 10-fold cross validation the standard deviations for the measures across the folds are given in parentheses in the table. We see that model performs relatively well with an AUC of 0.69 and accuracy of 0.7 for all ages.

**Table 9:** Discrimination and calibration metrics when the model is tested on all ages and by age group.

	**All ages**	**less than 50**	**50-59**	**60-69**	**70 plus**
AUC	0.69 (0.03)	0.52	0.64	0.69	0.69
F1	0.42 (0.03)	0.31	0.43	0.37	0.49
Accuracy	0.70 (0.70)	0.64	0.66	0.69	0.73
Hosmer and Lemeshow test	0.26 (7)	3.4e-5	0.03	0.44	0.12
Spiegelhalter's test	0.54 (9)	0.002	0.09	0.24	0.38

To break the discrimination metrics down further we look at how well the model predicts for individual age groups, by finding the metrics for the individual age group data sets. While the model seems to predict reasonably well for the older age groups, the AUC, F1 measure, and accuracy drop considerably for the model when it is tested on the less than 50 age group and we also observe a drop in the AUC when tested on the 50–59 age group.

##### 3.3.1.2. Calibration

Table 9 also shows the *p*-values for the Hosmer and Lemewshow goodness of fit test and Spiegelhalter's *Z*-test. As we do a 10-fold cross validation the *p*-values presented in the table are the average *p*-value across the 10-folds and the number of folds where the *p*-values are not significant is included in brackets. The table also includes the *p*-values for the individual age groups. For the all ages data set, both *p*-values are not significant, which suggests that on the whole the model might be well-calibrated. We also include the number of folds where the *p*-values were not significant, and can see that for 7 out of 10-folds the Hosmer-Lemeshow test suggests good calibration and for 9 out of 10-folds the Speigelhalter's *Z*-test suggests good calibration.

From Table 9, we also can see that while the model appears to be well-calibrated in the older age groups it is not well-calibrated in the younger age groups. Combined with the under discrimination measures, the model trained on data across all ages does not predict well, nor is it well-calibrated, for the younger age groups.

#### 3.3.2. Separate Risk Models by Age Group

We then fit separate multi-variable logistic regression models by age group. These models have the same risk factors that are included in the model trained on all age groups with the exception of age.

Looking at the coefficients and the statistical significance of the coefficients in Table 10 we observe some interesting patterns. We see the coefficient for systolic blood pressure tends to decrease in size as age increases going from 0.57 in the less than 50 age group to 0.15 in the 70 plus age group. Diastolic blood pressure increases from a non-significant coefficient of 0.09 in the less than 50 age group to 0.25 in the 70 plus age group, with the highest magnitude of the coefficient in the 50–59 age group. We also see other increases in magnitudes in the coefficient for atrial fibrillation, from 1.18 in the less than 50 group to 2.68 in the 70 plus group.

**Table 10:** Coefficients for the age group specific logistic regression models (*indicates a scaled variable).

	**Less than 50**	**50–59**	**60–69**	**70 plus**
Intercept	−0.87	−1.05	−0.95	−0.95
	0.0009	3.4e-11	<2e-16	<2e-16
Sex	0.05	0.14	−0.15	0.12
	0.87	0.48	0.24	0.20
Systolic blood pressure*	0.57	0.20	0.31	0.15
	0.04	0.17	0.0007	0.0008
Diastolic blood pressure*	0.09	0.42	0.24	0.25
	0.71	0.006	0.009	1.12e-07
BMI*	0.21	0.17	0.02	0.05
	0.24	0.07	0.76	0.28
Cigarettes smoked per day	0.003	−0.007	0.03	0.02
	0.77	0.35	1.48e-07	0.0003
Atrial fibrillation	1.18	1.78	2.28	2.68
	0.07	0.0001	7.00e-16	<2e-16
Diabetes	−0.42	1.04	1.12	0.21
	0.64	0.0004	1.43e-09	0.17

Besides changes in magnitudes we also see changes in significance of coefficients between age models for the same variables. The number of cigarettes smoked per day is significant in the older age groups, 60–69 and 70 plus, but not for the younger age groups, less than 50 and 50–59. Diabetes is significant in the middle age groups, but not in the oldest and youngest age groups. BMI is only significant in the 50–59 age group which is also the only age group (in this case at the 10% level) where systolic blood pressure is not significant.

##### 3.3.2.1. Discrimination

We evaluate the age models using the same methodology we applied to evaluate the model trained on all ages, looking at both discrimination and calibration. Table 11 shows the discrimination results presenting the AUC, F1 measure, and accuracy for each model from a 10-fold cross validation and the standard deviation for each measure in parentheses. We see higher AUCs for all age groups compared to the single all ages model with age as a risk factor and higher accuracy for the two younger age groups. While the F1 measure drops in a few of the age groups, the higher AUC might suggest that a change in the prediction threshold may lead to better predictions.

**Table 11:** Discrimination and calibration metrics for each age model.

	**Less than 50**	**50–59**	**60–69**	**70 plus**
AUC	0.67 (0.16)	0.70 (0.05)	0.72 (0.03)	0.70 (0.03)
F1	0.22 (0.16)	0.41 (0.09)	0.47(0.06)	0.44(0.04)
Accuracy	0.70 (0.10)	0.71 (0.05)	0.68 (0.04)	0.71(0.02)
Hosmer and Lemeshow test	0.23 (7)	0.34 (9)	0.32 (8)	0.35 (10)
Spiegelhalter's test	0.34 (7)	0.52 (9)	0.44 (7)	0.58 (10)

##### 3.3.2.2. Calibration

We also look at the calibration of the models. Table 11 also shows the average *p*-value for the Hosmer and Lemeshow test, and the average *p*-value for the Spiegelhalter's *t*-test across the 10-folds of model validation. We also include the number of folds that result in a “well-calibrated” model based on the Hosmer and Lemeshow test and then again based off of the Spiegelhalter's test in parentheses.

None of the *p*-values in the table are significant suggesting all four age models are well-calibrated. For both tests the 70 plus group was well-calibrated in all 10-folds, the 50–59 group was well-calibrated in 9-folds and the less than 50 group in 7-folds. The 60–69 age group was well-calibrated based on the Hosmer Lemeshow test in 8-folds and 7 based on the Spiegelhalter's test. Compared to the *p*-values in Table 9, we get better calibration when modeling by age group especially in the younger age groups.

##### 3.3.2.3. Feature Importance

While we see that coefficient size and significance of the coefficients change between age groups with some patterns forming, to further look at the differences in risk factors between age groups we look at the feature importance ranking for each of the age models.

**Figure 1:**
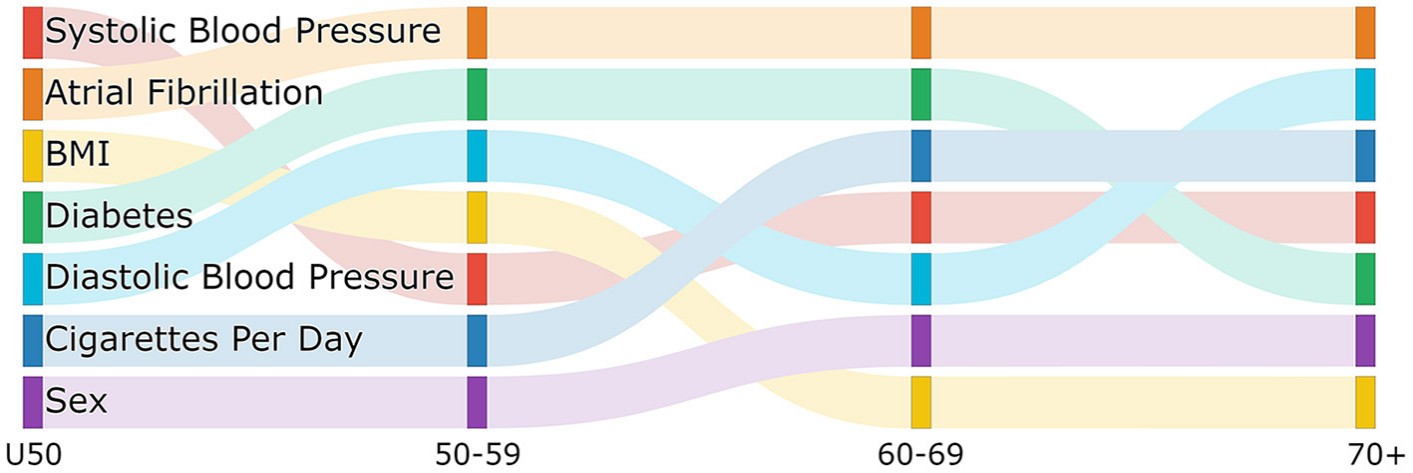
Sankey diagram showing the change in feature importance ranking by age group.

Figure 1 is a sankey diagram showing how the features change in importance across the age group models. Each column represents a different age model and each row in the column represents a feature with the top row being the most important feature. Following the colored links through the diagram shows how the feature importance changes between models. Although we see some features that have high importance across all age models, such as atrial fibrillation, we also see some changes in importance. For example, while BMI is the 3rd and 4th most important feature in the less than 50 and 50–59 group respectively it drops to the 7th for the two older age groups. We also see that diabetes is more important in the middle two age groups compared to the older and younger groups, and the average number of cigarettes per day is more important in the two older age groups than the younger two age groups.Interestingly, the importance of systolic blood pressure and diastolic blood pressure appear to change with age. While systolic blood pressure is more important in the under 50 and 60–69 age group, diastolic blood pressure is more important in the 50–59 and 70 plus age group. In the figure, we can also see where there are changes in the rank of important features moving between age groups which might give an indication of where the interactions of risk factors and age might change. For example, the rank of importance of cigarettes per day and sex are the same for the under 50 and 50–59 age group and then change in importance for the 60–69 age group but are the same between 60–69 and 70 plus. This might indicate that there is a biological change between the 50–59 age group and the 60–69 age group that leads to this difference in risk factor importance.These changes are showing that features contribute differently to the risk of each age group, further supporting the needs for separate models by age group.

## 4. Discussion

Age is a key factor in an individual's stroke risk with the probability of having a stroke increasing with age. However, there are other risk factors that are important in determining the risk of stroke by age and if age is considered as a risk factor in a model we have shown that the model may neglect the contribution of these other factors. Additionally, the importance of risk factors and contributions of different risk factors to an individual's risk changes with age. To account for the differences in importance and contribution by age while still accounting for the contribution of age we have created a set of models that predict individual risk of stroke by age group. We have shown that when looking at both discrimination and calibration metrics, the age models are able to predict better and are better calibrated, particularly in the younger age groups. While we do not see a huge difference in the predictions for the 60–69 group and the 70 plus group, the increase in prediction, accuracy, and calibration for the less than 50 and 50–59 group may help to identify younger individuals with a high risk of stroke who may not be identified using a traditional stroke risk prediction model.

The age models allow us to look at risk factors and their changing contribution and difference in importance by age. Although stroke is often seen as a health event, there are a number of processes in the etiology that have lead up to that event (20). Identifying the risk factors and interactions between risk factors that change in importance between age groups cannot only provide us with a better short term risk prediction but may help to identify those who might have a high long term risk despite a low short term risk because of the impact of a risk factor over a long period of time. For example, while diabetes is not seen as an important risk factor in those less than 50 it becomes more important in the older age group and while a 45 year old with diabetes and no other major risk factors might not have a high short term risk of an ischemic stroke, their long term risk might be higher. Understanding how the importance of risk factors change with age may be essential in lowering the overall burden of stroke on society.

Future work can be done to add in additional features, for example lifestyle factors such as exercise levels or community involvement. These risk factors may help to identify those who are more at risk for a stroke in the short term and are factors that are likely to change in importance with age. Additionally, in this paper we focus on if differences occur between the distributions of risk factors by age group and how those differences impact modeling. We do not do an in depth analysis of the impact of individual risk factors by age or research into why one risk factor might be more important in one age group over another. Future work can focus on a more in depth explanation of risk factors according to age to better understand why we see the variations found in this work.

Some of the limitations of the study include that the models presented here predict short term risk, within 5 years, of an ischemic stroke. However, while still important for those with a high short term risk, long term risk, especially for the younger population with generally low short term risk, might be more informative. In predicting longer term risk the age models may prove more important in identifying those features that might not contribute highly to stroke risk when under a given age but become more important as an individual ages. Additionally we have the potential of looking at different time windows for different age groups. For example, in the less than 50s, 10 or 20 year risk might be a more useful time frame, whereas it might be more important to predict the 5 year risk for the 70 plus age group. Predicting risk by age group allows us to look at different time horizons for different age groups. The statistical analysis and the creation of the models were all done using data from the Framingham Heart Study. While we believe that the non-proportionality of risk factors by age found here is likely to apply to other populations, it might be necessary to analyze other populations to determine that the patterns found here are not a characteristics of the specific population. Other populations might have different distributions of risk factors that could impact the proportionality or non-proportionality of those risk factors by age. It might be necessary to re-calibrate the model to another population's data to get the best risk predictions for individual's in a new population. Further while creating separate risk models by age group appears to produce models that are better calibrated, the method has the limitation of reducing the amount of data used to train each model. If the sample size is already small breaking it into age groups could result in sample sizes too small to be confident in any effects captured by the model. Finally, while we use logistic regression models here, other methods for risk prediction may be used. For example, survival analysis, neural networks, or Poisson regression.

## Data Availability Statement

The data analyzed in this study was obtained from the National Institute of Health (NIH) Biologic Specimen and Data Repository Information Coordinating Center (BioLINCC) website, the following licenses/restrictions apply: these datasets must be requested from the NIH BioLINCC website. Requests to access these datasets should be directed to https://biolincc.nhlbi.nih.gov/studies/gen3/.

## Author Contributions

EH and JK: conceptualization and methodology. EH: formal analysis and writing—original draft preparation. JK: writing—review and editing and supervision. All authors have read and agreed to the published version of the manuscript.

## Funding

This project received funding from the EU's Horizon 2020 research and innovation programme under grant agreement No. 777107, and by the ADAPT Centre for Digital Content Technology funded under the SFI Research Centres Programme (Grant 13/RC/2106_P2) and co-funded under the European Regional Development Funds.

## Conflict of Interest

The authors declare that the research was conducted in the absence of any commercial or financial relationships that could be construed as a potential conflict of interest.

## Publisher's Note

All claims expressed in this article are solely those of the authors and do not necessarily represent those of their affiliated organizations, or those of the publisher, the editors and the reviewers. Any product that may be evaluated in this article, or claim that may be made by its manufacturer, is not guaranteed or endorsed by the publisher.

## Footnotes

^1^For more information on the data and data collection, see https://biolincc.nhlbi.nih.gov/home/.

^2^The Brier Score is the mean squared error between the outcome and the estimated probabilities.
